# Spatial and Temporal Entropies in the Spanish Football League: A Network Science Perspective

**DOI:** 10.3390/e22020172

**Published:** 2020-02-02

**Authors:** Johann H. Martínez, David Garrido, José L. Herrera-Diestra, Javier Busquets, Ricardo Sevilla-Escoboza, Javier M. Buldú

**Affiliations:** 1 Biomedical Engineering Department, Universidad de los Andes, 111711 Bogota, Colombia; 2 Grupo Interdisciplinar de Sistemas Complejos (GISC), 28911 Madrid, Spain; 3 Complex Systems Group, Rey Juan Carlos University, 28933 Madrid, Spain; 4 Laboratory of Biological Networks, Centre for Biomedical Technology (CTB-UPM), Universidad Politécnica de Madrid (UPM), 28223 Madrid, Spain; 5 ICTP—South American Institute for Fundamental Research, 01140-070 Sao Paulo, Brazil; 6 CeSiMo, Facultad de Ingeniería, Universidad de Los Andes, 5101 Merida, Venezuela; 7 Department of Operations, Innovation and Data Science, ESADE Business School, 08034 Barcelona, Spain; 8 Centro Universitario Los Lagos, Universidad de Guadalajara, 47463 Lagos de Moreno, Mexico; 9 Institute of Unmanned System and Center for OPTical IMagery Analysis and Learning (OPTIMAL), Northwestern Polytechnical University, Xi’an 710072, China

**Keywords:** football, spatial entropy, network science, permutation entropy, statistical complexity, team performance

## Abstract

We quantified the spatial and temporal entropy related to football teams and their players by means of a pass-based interaction. First, we calculated the spatial entropy associated to the positions of all passes made by a football team during a match, obtaining a spatial entropy ranking of Spanish teams during the 2017/2018 season. Second, we investigated how the player’s average location in the field is related to the amount of entropy of his passes. Next, we constructed the temporal passing networks of each team and computed the deviation of their network parameters along the match. For each network parameter, we obtained the permutation entropy and the statistical complexity of its temporal fluctuations. Finally, we investigated how the permutation entropy (and statistical complexity) of the network parameters was related to the total number of passes made by a football team. Our results show that (i) spatial entropy changes according to the position of players in the field, and (ii) the organization of passing networks change during a match and its evolution can be captured measuring the permutation entropy and statistical complexity of the network parameters, allowing to identify what parameters evolve more randomly.

## 1. Introduction

In the recent years, the analysis of organization and performance of both football teams and their players underwent a revolution [1,2,3,4,5]. The main reason is the access to new sets of data thanks to the application of emergent technologies to the recording of players’ activity during a match [6]. In this way, it is possible to have access to all events occurring on the pitch (passes, interceptions, shots, goals, fouls, etc.), all of them with precise temporal and spatial coordinates, and the players responsible for each event. On the other hand, it is also possible to track the positions of all (twenty-two) players on the field, together with the ball, at rates approaching 25–30 frames per second [7,8], which allows one to determine the position, speed and acceleration of each player, giving very useful information about their physical and tactical performances [9,10,11].

However, the key advances have not been the amount and quality of the datasets themselves, but the possibility of applying (or defining) new methodological tools. On that sense, the application of network science [12] to football datasets is giving a completely new perspective about football analysis, since it allows one to understand the roles of players as a whole, not as isolated components without interactions between them. In this way, it is possible to construct *football passing networks*, composed of nodes (players) and links (passes between players), whose organization is far from being random. The analysis of football passing networks has shown that their properties continuously evolve during a match and that key events, such as goals, may affect the network organization [13]. Classical network properties, such as the clustering coefficient [14], the shortest path length [15] and the existence of motifs [16] or communities of players with strong interactions between them, have been investigated [17], allowing not only the characterization of the organization of a team but also quantification of the roles of players in the network [18,19].

In this paper, we are concerned with one particular feature of football passing networks: Entropy. Specifically, we are interested in understanding how the network organization fluctuates along a match, and identifying those network parameters that evolve in a more (or less) random or complex manner. The reasons are twofold. One the one hand, detecting those parameters that behave more randomly should give us useful information about their (low) predictability, and, at the same time, should show us which parameters behave less randomly, suggesting that certain guiding rules could be behind their evolution. On the other hand, we determine to what extent the features of football teams (in this case, the entropy of the network parameters) are general, whether they change from team to team and what their relations to team performance are.

Importantly, previous studies about the entropy existing in football have left the existence of an underlying network of connections aside. For example, the use of Generalized Entropy (GE) [20] to analyze the distribution of the number of points obtained by football teams at the end of a season allows quantifying the competitive balance of a given league [21]. As a result, it was shown that the competitiveness of English Premier League (EPL) is highly imbalanced, and different noncompeting groups were identified: (i) the top five teams aspiring to win the league or to reach the European Championship, (ii) a group of teams competing for positions in lesser European competitions and (iii) a set of teams just aspiring to maintain a position in the EPL. A completely different approach was made by Sampaio and Maçãs, who used the Approximate Entropy (AE) [22] to quantify the regularity levels of the movements (measured with GPS devices) of players with different levels of expertise [10]. The approximate entropy allowed them to evaluate the in-phase and anti-phase coordination of players, showing more regular patterns after a 13-week football program. A posterior study computing the AE during the development of a match showed that AE decreases as time goes by, indicating a tendency toward more regular patterns that may be attributed to players’ fatigue [23]. It is also possible to relate the level of entropy of players’ movements to their position in the field. In [8], the Shannon Entropy (SE) and the AE computed over the heat maps of players during a match showed that goalkeepers are the players with the lowest entropy, while midfielders are those with the highest.

Interestingly, entropy is also dependent on the number of interacting players, as shown by [11] in the context of small-sided games. These kinds of exercises involve a reduction of the pitch dimensions together with a low number of players, which can change depending on the kind of game. The computation of the Boltzman-Gibbs-Shannon entropy [24] to the position of players showed that the larger the number of opponents, the higher the regularity of the movements of a team (as shown by a reduction of their entropy) [11].

In view of all, entropy in football has been mainly associated to the position of players [25]; however, the recent advances made by the application of network science to football datasets open the door to an alternative perspective: The evaluation of the entropy related to passing networks, and more specifically, the understanding of the fluctuations of their structure along time. With this aim, we constructed the temporal passing networks obtained from the football matches of Spanish national league (“La Liga”) during the season 2017/2018. We observed how the structure and parameters of *time-evolving passing networks*, which were constructed using the last 50 passes made by a team at every moment of the match, have continuous fluctuations, and we analyzed both the entropy and complexity levels of these fluctuations using ordinal patterns, a technique that has been demonstrated to extract hidden patterns from real time series [26]. Our results allowed us to determine which network parameters have higher/lower entropy; which teams behave more randomly; and finally, what the interplay is between the average number of passes made by a team and the entropy/complexity of the resulting passing networks.

## 2. Results

### 2.1. Datasets

Datasets were provided by Opta [27] and consist of all passes completed in a football match by each team of the first division of the Spanish national league (“La Liga”) during the season 2017/2018. Specifically, consists of a set of 380 matches, 38 per team. For all completed passes, we have the information about: (i) the player who passed the ball, (ii) the player who received the ball, (iii) the positions (*x* and *y* coordinates) of the sender and the receiver and (iv) the time at which the pass was made (see Table 1 for details).

We obtained a total of 284,813 completed passes from the 380 matches, with a total number of passes per match ranging from 442 (Eibar vs. Leganés) to 1082 (Real Madrid vs. Real Sociedad). Concerning the differences of length and width existing between all pitches of the league, the x and y coordinates are given in “field units,” bounded at both axis between 0 and 100. The x coordinate increases as we are approaching the opponent’s goal, while the coordinate y=0.0 corresponds to the left limit of the pitch and y=100.0 to the right one. In this way, the coordinate (50.0, 50.0) locates the center of the pitch, the opponent’s goal is centered at (100.0, 50.0) and a team’s own goal is centered at (0.0, 50.0) Finally, when a substitution occurs, the incoming player takes the place (as a node of the network) of the replaced player. That way, we place the focus on what a team is doing instead of looking at the roles of the players.

### 2.2. Spatial Analysis: Quantifying Spatial Entropy

First, we quantified the heterogeneity of the positions of all passes completed during the match, with the aim of determining whether the passing positions are randomly distributed, or on the contrary, follow a certain pattern. Specifically, we only considered the position from where each completed pass was initiated. From the diversity of metrics quantifying the spatial randomness of a distribution of discrete objects, we used the methodology proposed by Clark and Evans [28], since it combines the concepts of clustering, randomness and regularity, all of them with a straightforward interpretation within the framework of a football match. Therefore, for a set of *n* points (passes) spatially distributed over a two-dimensional space, we computed the distance $r_{i,j}$ between a point *i* and its nearest neighbor *j*. Next, we obtained the mean nearest neighbor distance $\langle r\rangle =\frac{1}{n}\sum_{i\neq j}^{n}r_{i,j}$. Note that complete spatial randomness of *n* points distributed over a surface *S* is described by a Poisson process (which, as we will see, it is not the case for a football match), in which the probability density function for the nearest neighbor distance, *r*, is $p\left(r\right)=2\pi \delta re^{\left(-\pi \delta r^{2}\right)}$, with δ=n/S being the point density. If we assume that each point has the same probability of appearing at any position on the surface *S*, the expected average distance between nearest neighbors is given by $r_{ran}=\frac{1}{2}\sqrt{S/n}$ [28].

Normalizing the reported mean nearest neighbor distance by the one expected in a random distribution, we obtain the *spatial entropy parameter* $H_{s}=\frac{\langle r\rangle }{r_{ran}}$, which measures how far the real distribution of passes is from a completely random one. Interestingly, values of $H_{s}$ far from one give additional information. For example, $H_{s}$ approaching zero reveals that the mean distance to the nearest neighbors is very low, which is a consequence of the existence of clusters; i.e., small regions of the field containing a high number of passes. On the contrary, values of $H_{s}$ higher than the unity are related with situations where the positions of the passes are more separated than in a random distribution. For example, the largest value of $H_{s}$ in a two-dimensional space occurs when points are placed in a triangular lattice arrangement, leading to $H_{s}^{max}=2.149$. This way, the closer the value of $H_{s}$ is to $H_{s}^{max}$, the more regular the distribution of passes is.

**Figure 1:**
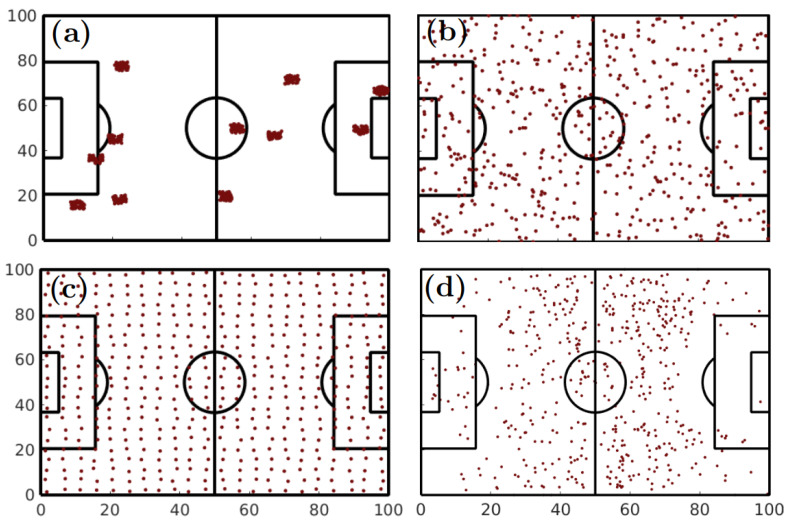
Spatial entropy parameter $H_{s}$. (**a**–**c**) Three examples of n=517 artificially generated passes for which the distances between locations where passes were made were increased. (**a**) Passes form clusters, leading to a low spatial entropy parameter ($H_{s}=0.14$). (**b**) The location of the passes is randomly generated, resulting in a spacial entropy very close to one ($H_{s}=1.01$). (**c**) The distance between nearest neighbors is close to its optimal, leading to a regular spatial distribution of passes and a spatial entropy ($H_{s}=1.73$) close to the highest possible value in 2-dimensional distributions. Finally, (**d**) the real location of all passes made by Real Madrid during its match against F.C. Barcelona (season 2017/2018). We obtained a value of ($H_{s}=0.88$), revealing that the actual distribution of passes is close to the random case.

Figure 1 shows four different examples of the spatial entropy parameter $H_{s}$ in the context of football passes. All plots of Figure 1 contain the same number of passes (n=517). However, plots in Figure 1a–c were artificially generated, while Figure 1d corresponds to a real case: passes made during the match between Real Madrid and F.C. Barcelona during the season 2017/2018. In Figure 1a the spatial entropy parameter is $H_{s}=0.14$ and corresponds to the cases where passes are grouped in clusters, leading to a low distance to nearest neighbors. Figure 1b shows the case where points are randomly distributed, resulting in a value of $H_{s}=1.01$. In Figure 1c, all passes are placed close to the configuration where the distance to the nearest neighbors is maximum, leading to $H_{s}=1.73$. Finally, Figure 1d shows the actual locations of all passes made by Real Madrid during its match against F.C. Barcelona, where $H_{s}=0.88$. As we can see, the spatial entropy parameter has a value that is very close to the random case.

**Figure 2:**
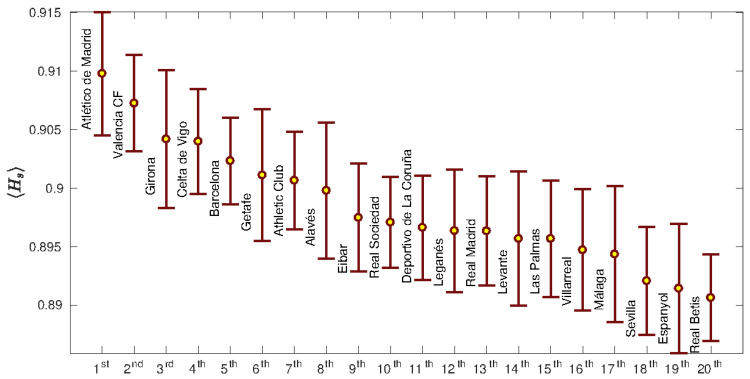
Values of the spatial entropy parameter $H_{s}$ for all Spanish teams during the season 2017/2018. Error bars are the standard deviations of the means. Atlético de Madrid was the team with the highest spatial entropy parameter (∼0.91), in contrast with Real Betis, which had the lowest one (∼0.89). Note that all values are slightly lower than the unity, indicating a high randomness for the locations of the passes.

To see how general the example shown in Figure 1d is, we computed the average spatial entropy of each team, obtained for the matches played during the whole season. Figure 2 shows the $H_{s}$ ranking of all teams, which in all cases is slightly below one. These results indicate that the locations of the passes made during a football match are highly random, no matter what team is playing (see in Figure 2 how all values are close to one). Furthermore, the deviation of the average values of the spatial entropy is not very high and all spatial entropies are contained within the interval (0.89,0.91). Interestingly, the team that won the league that season, F.C. Barcelona, was in the fifth position of the ranking, and furthermore, we did not report any correlation between the number of points at the end of the season and the average spatial parameter.

**Figure 3:**
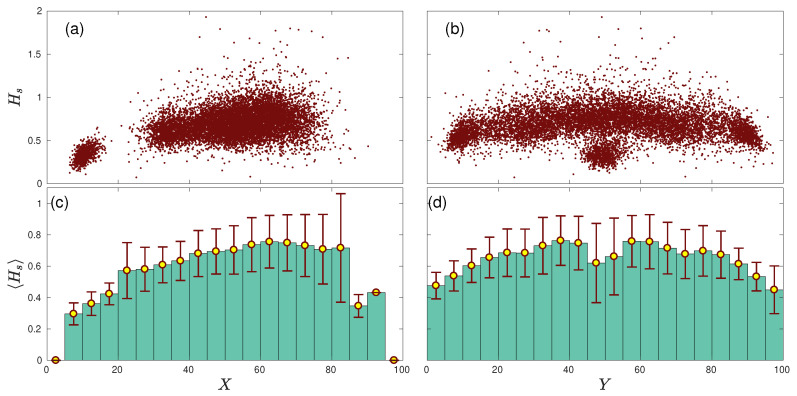
Spatial entropy $H_{s}$ vs. the location of the players. (**a**) Each dot depicts the spatial entropy for the average x-coordinate of the passes made by players during each match. (**b**) Here, the absolute entropy is plotted as a function of the y-coordinate of players. (**c**) Average spatial entropy $\langle H_{s}\rangle$ in 20 subdivisions of the field (with a width of 5 field units) along the whole season. Errors bars are the standard deviations of each subdivision. (**d**). Same as (**c**) but considering the Y axis.

The spatial entropy of a team is the result of a collective phenomenon based on the interaction between players—in this case, exchanging the possession of the ball between them. Therefore, our next step is having a look at a lower scale and investigating what the spatial entropy is corresponding to the passes made by each player. In Figure 3a,b we plot the spatial entropy $H_{s}\left(i\right)$ of player *i*, computed for every match, together with the average position X(i) and Y(i) of all passes made by the player during the match. Both *X* and *Y* are bounded between (0, 100). For the *X* coordinate, 0 corresponds to the closest location to the player’s own goal and 100 to the rival goal. For the *Y* coordinate, 0 corresponds to the left limit of the pitch and 100 to the right one. Note that we are using the average location of all passes made by a player as a proxy to estimate his average position in the field.

This representation shows the interplay between the spatial entropy of the passes made by a given player and his position in the field. Interestingly, Figure 3a shows two clear clusters of points. The cluster on the bottom left corresponds to the spatial entropy of goalkeepers, who have a low spatial entropy due to the fact that the locations of their passes are clustered close to the goal (i.e., goalkeepers always try to avoid moving from their goals). The second cloud of points corresponds to the rest of players, and their spatial entropy shows a slight tendency to increase as the player is close to an opponent’s goal. Figure 3c shows the same information where the average of the spatial entropy every 5 field units $\langle H_{s}\rangle$ is calculated. We can observe how $\langle H_{s}\rangle$ increases with the position of the player until X=60; beyond this point, it seems to saturate, while at the same time, the error bars increase. Finally, the entropy decreases for positions very close to the opponent’s goal, which seems to be a consequence of the low number of passes within these regions.

Figure 3b,d show how the spatial entropy depends on the *Y* coordinate; i.e., the lateral position of the player. We can also observe the cloud corresponding to the goalkeepers centered around Y=50, which corresponds to the lowest values of spatial entropy. Disregarding the goalkeepers, we can observe a slight decrease of the spatial entropy as the players move to the sides of the field. This way, players playing in the center of the pitch have higher spatial entropies. This fact is hidden in Figure 3d, since the average entropy $\langle H_{s}\rangle$ around position Y=50 is strongly reduced by the low values related to goalkeepers.

**Figure 4:**
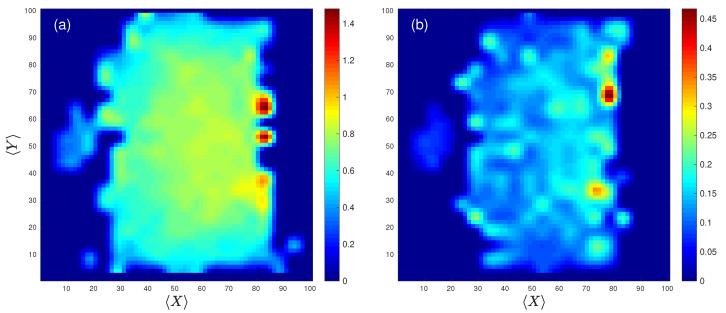
Spatial entropy parameter $\langle H_{s}\rangle$ vs. location on the pitch. (**a**) Spatial entropy parameter $\langle H_{s}\rangle$ averaged over all players. (**b**) The corresponding standard deviations of the values shown in (**a**).

Finally, Figure 4 shows a two-dimensional plot of both the average spatial entropy $\langle H_{s}\rangle$ (a) and its corresponding standard deviation (b) calculated for all players during the whole season. On the center-left, we can observe an island containing the values of the spatial entropy of goalkeepers and how, for the rest of players, spatial entropy slightly increases while approaching the opponent’s goal. In that regard, Figure 4b shows that the highest values of the standard deviation appear in the attacking regions, which may be interpreted as a signature of the high variability that forward players have in their playing patterns.

### 2.3. Temporal Entropy and Complexity of Football Passing Networks

Arriving at this point, we payed attention to the entropy related to the organizational properties of teams and how the network of interactions between players evolved. Thus, we analyzed the structure of the passing networks associated to each team and how their parameters fluctuated during a match. As explained in the introduction, the nodes of the passing networks are the players of a team and links (weighted and directed) arising from the number of passes made from one player to any other [17]. Note that, in this case, entropy should capture the *temporal* fluctuations of the network parameters. We constructed the “*50-pass networks*” at every moment of the match, each of them containing the last 50 consecutive passes. This way, after the beginning of the match, we selected the first l=50 passes of a team to construct, for each team, the initial passing network $G_{1\to 50}$. The nodes of this network are the players and the links contain the directions of and the numbers of passes between pairs of players, leading to a directed and weighted network. To ease the comparison between networks of different teams, each titular player was assigned a node at the beginning of the match. If a player was substituted, the new player occupied the node of the previous player. That way, we assured that all networks had eleven players, focusing on the structure of the network as a whole instead of the performance of isolated players. Next, each time a new pass was made, we disregarded the oldest pass of the previous network and included the new one, assigning the time *t* of the last pass to the new network. This kind of temporal network has two advantages: (i) it allows accounting for the fluctuations of the network parameters along the match and (ii) it has exactly the same number of nodes and links for any team, which detaches the influence of the absolute number of passes and focuses only on the structural differences between networks. The number *l* of passes used to construct the network could be modified to another value; however, it should be low enough to account for the fluctuations occurring during the match (i.e., avoiding averages) and long enough to guarantee the connectivity between all nodes of the network. In our case, we analyzed the effects of using different number *l* of passes and chose l=50 as a trade-off value.

For each temporal network of each team, we computed a group of spatial parameters; namely: (i) the *x*-coordinate of the network centroid $X_{CM}$, (ii) the *y*-coordinate of the network centroid $Y_{CM}$ and (iii) the spatial dispersion $\sigma_{CM}$ of the players around the network centroid (in field units). Next, we calculated a second group of parameters related, in this case, to the organization of the temporal passing networks: (i) the weighted clustering coefficient *C*; (ii) the shortest path length sp; (iii) the largest eigenvalue of the adjacency matrix $\lambda_{1}$, which is an indicator of the network strength [29]; (iv) the largest eigenvector centrality of a player $ec_{max}$ (measuring the existence of a leading player); and (v) the dispersion of the centrality of all players of the team $\sigma_{ec}$. We also computed the average time required by each team to construct 50-pass networks $\Delta t_{50}$. See Section 4.1.1, Section 4.1.2, Section 4.1.3, Section 4.1.4 and Section 4.1.5 of Methods for a detailed description of each parameter and the way it was obtained.

**Figure 5:**
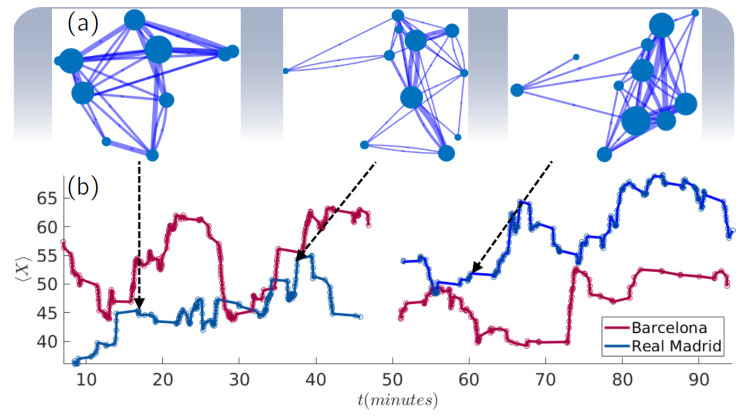
Evolution of passing networks and their corresponding parameters. (**a**) The structure of Real Madrid 50-pass network at three different moments of the match ($t_{1}$ = 15:00, $t_{2}$ = 38:00 and $t_{3}$ = 60:00). (**b**) An example of how a network parameter fluctuates during the match. Specifically, we plotted the $X_{CM}$ of Real Madrid (blue) and F.C. Barcelona (red), the latter playing at home.

Figure 5 shows an example of how network parameters fluctuate during a match. In our case, we plotted the evolution of the *x*-coordinate of the 50-pass network centroid $X_{CM}$ of Real Madrid and F.C. Barcelona during the match played at *Camp Nou* Stadium. In Figure 5a, we also schematically show the network configuration of Real Madrid at three different moments of the match ($t_{1}=$ 15:00, $t_{2}=$ 38:00 and $t_{3}=$ 60:00). In these plots, nodes represent the players that interacted in the corresponding temporal networks. Their positions were obtained as the average coordinates from which their passes were made. Concerning the width of a link, it is proportional to the number of passes made between the two players the link connects. We can observe how the fluctuations of the value of $X_{CM}$ are related not only to the players’ positions, but also to a reorganization of the passing network.

With the aim of understanding how the network parameters fluctuate, we computed the permutation entropy (PE) [30] and the statistical complexity (SC) [31] of the time series corresponding to each of the network parameters. Both variables (PE and SC) were measured by means of the methodology proposed by Bandt and Pompe [30], which quantifies temporal aspects of time series thanks to a translation to a set of symbols. This methodology has been proven to be fast, robust to noise and effective for weak stationary processes [31,32,33], and has been applied in many different disciplines, such as ecology, lasers, nonlinear dynamics, biomedicine, neuroscience and economics [26,33,34,35,36,37]. This method is also capable of capturing the entropy and complexity along several dimensions. (See Section 4.2 of Methods for a detailed explanation about how PE and SC are calculated). Note that in this section, entropy (and complexity) refer to temporal fluctuations and not to the spatial ones. Therefore, we called the temporal entropy $H_{t}\left(p\right)$ of a network parameter *p* to the PE of the time series of that parameter *p*, while the temporal complexity $SC_{t}\left(p\right)$ was obtained by calculating the corresponding SC.

**Figure 6:**
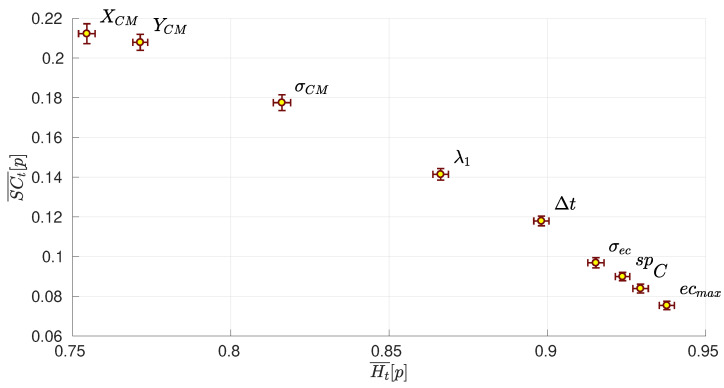
Complexity-entropy diagram of all network parameters. Each point represents the average value of the entropy $\bar{H}\left[p\right]$ and complexity $\bar{SC}\left[p\right]$ of each network parameter *p* for all the 20 teams of “*La Liga*” during the season 2017/2018. Error bars are the standard deviation of the means. Specifically, network parameters are: the *x*-coordinate of the network centroid $X_{CM}$, the *y*-coordinate of the network centroid $Y_{CM}$, the spatial dispersion $\sigma_{CM}$ of players around the network centroid, the clustering coefficient *C*, the average shortest path length sp, the largest eigenvalue of the adjacency matrix $\lambda_{1}$, the largest eigenvector centrality of a player $ec_{max}$ and the dispersion of the centrality of all players of the team $\sigma_{ec}$.

Figure 6 shows the 2-dimensional plot of the complexity vs. entropy of each network parameter, which has been computed by averaging the values of $H_{t}$ and $SC_{t}$ obtained for the 380 matches played during the season. Note that for each match, we have two values (one for each team) for each parameter. We can observe how the network parameters related to the coordinates of the network’s centroid ($X_{CM}$ and $Y_{CM}$) are those parameters with the lowest entropy, and conversely, with the highest complexity. This fact indicates that both $X_{CM}$ and $Y_{CM}$ are the parameters that evolve less randomly, and therefore, are good candidates for making predictions about the evolution of the network. On the other hand, we can observe how the highest centrality of a player $ec_{max}$ is the parameter with the highest entropy. Note that the centrality dispersion $\sigma_{ec}$ also has a high entropy. Both facts indicate that the evolution of the team centrality behaves rather randomly. Interestingly, the evolutions of two classical network parameters, the average shortest path sp and the clustering coefficient *C*, are quite random, as indicated by their high entropy and low complexity.

**Figure 7:**
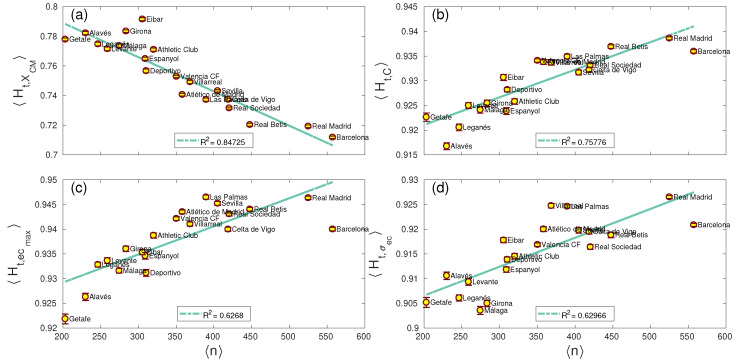
Temporal entropy $H_{t}$ vs. average number of 〈n〉. Only network parameters with a correlation $r^{2}>0.6$ are shown; namely, the *x*-coordinate of the network centroid $X_{CM}$, the clustering coefficient *C*, the largest eigenvector centrality of a player $ec_{max}$ and the dispersion of the centrality of all players of the team $\sigma_{ec}$. (**a**) The average of $X_{CM}$ for each team along the whole season has a negative correlation with the number of passes ($R^{2}=0.74$). (**b**) The average clustering coefficient *C* has a positive correlation with $R^{2}=0.79$, as it is the case of $ec_{max}$ (**c**) and $\sigma_{ec}$ (**d**).

Finally, we analyzed the entropy (and complexity) related to each team. Specifically, we were concerned about the interplay between the average number of passes 〈n〉 made by a team and the resulting entropy and complexity. Figure 7 shows those network parameters whose average entropy $\langle H_{t}\rangle$ vs. 〈n〉 had a correlation parameter fulfilling $r^{2}>0.6$ (see the same results for the complexity $\langle SC_{t}\rangle$ vs. 〈n〉 in Appendix A). Interestingly, only the *x*-coordinate of the network centroid $X_{CM}$, the clustering coefficient *C*, the highest centrality of a player $ec_{max}$ and the centrality dispersion $\sigma_{ec}$ had significant correlations with the average number of passes. This fact indicates that these parameters are indeed influenced by the number of passes made by a team, suggesting the coaching staff of a team should increase/decrease the number of passes as a way to modify the entropies of these parameters.

## 3. Discussion

Summarizing, we have investigated the spatial and temporal entropies of football teams, focusing on the locations of the passes made during a match and the evolution of the organization of each corresponding passing network. The 380 matches of the Spanish national league (“*La Liga*”) played during the 2017/2018 season were analyzed. First, we observed that the spatial entropy $H_{s}$ obtained from the passes’ positions of all teams was always close to the one, indicating a high randomness. Atlético de Madrid and Valencia C.F. are the teams with the highest average spatial entropy ($H_{s}=0.910$ and $H_{s}=0.907$, respectively), while R.C.D. Espanyol and Real Betis are the ones with the lowest entropy ($H_{s}=0.8914$ and $H_{s}=0.8906$, respectively). When the spatial entropy is analyzed at the level of the individual player, we observed differences related to the average position in the field. Goalkeepers are the players with the lowest spatial entropy, since they must remain close to their own goal, reducing their area of influence. On the other hand, the entropy of the rest of players increases as their position moves forward to the opponent’s goal. When the lateral distribution of entropy was analyzed, we observed higher entropies in the middle of the field, which decrease as the lateral boundaries are approached.

Next, we constructed the 50-pass networks of all teams and analyzed the fluctuations of their main parameters during each match. In this case, we computed both the permutation entropy and the statistical complexity, which account for temporal fluctuations of the time series of each network parameter once they are translated into ordinal vectors [26,30]. The the *x* and *y* coordinates of the center of mass of the network are the parameters with the lowest temporal entropies $H_{t}$ and the highest temporal complexities $SC_{t}$, revealing that randomness is not their main guiding rule. On the other hand, the highest eigenvector centrality of a player $ec_{max}$ and the clustering coefficient *C* are the network parameters with the highest entropy ($H_{t,ec_{max}}=0.938$ and $H_{t,C}=0.929$, respectively). Note that network parameters with the highest entropy are the ones whose prediction will be more complicated. Therefore, temporal entropy can be used to identify what parameters (those with the lowest values) are worth paying attention to, and to explore whether it is possible to actively modify them towards a given target.

Finally, we investigated whether the number of passes made by a team was related to the complexity/entropy of the network parameters. This is the case for the *x*-coordinate of the network centroid $X_{CM}$, the clustering coefficient *C*, the maximum centrality of a player $ec_{max}$ and the centrality dispersion $\sigma_{ec}$, which have correlation coefficients with average numbers of passes 〈n〉 higher than 0.6. Therefore, it is expected that increasing/decreasing the number of passes will result on a variation of the entropy/complexity of each of these parameters. Interestingly, the two referent teams of the Spanish league, Real Madrid and F.C. Barcelona have extreme positions in the ($H_{t},\langle n\rangle$) plot of these parameters (also in the ($SC_{t},\langle n\rangle$)). For example, both teams are the ones with the highest numbers of passes, and at the same time with the lowest entropy related to the *x*-coordinate of the network centroid. Since we observed a negative correlation between both parameters, a team aiming to reduce the entropy of its *x*-coordinate should try to increase its number of passes. The same reasoning, but in the opposite direction, can be applied to the other three network metrics. In this case, both *Real Madrid* and *F.C. Barcelona* are the teams with the highest entropy associated to *C*, $ec_{max}$ and $\sigma_{ec}$. Since the correlations between these parameters and the number of passes are positive, increasing the number of passes should lead to an increase of the entropy.

On the other hand, recent studies about *networks-of-networks* in other fields have shown that when networks get connected, important properties of the ensembled systems are modified [38,39]. With this regard, a multilayer description of a match, with two interacting networks composed of the internal passes of each team, is still missing. We think this could be a fundamental approach to understanding the evolution of entropy of the teams along the match, which cannot be interpreted without looking at the response of the opponent. The fact that the two networks are competing for a common resource and with an objective that directly implies interaction and competition with other networks, suggests new points of view about how networks behave, and particularly, how they generate entropy. When two teams (networks) are competing in the field, they need to develop strategies to create order/disorder, challenging the concept of the “interface” or dynamic limit between the two teams. In that regard, we should focus on how to create order and optimal organizational structures, but, at the same time, create “disorder” with the aim of generating situations of superiority.

However, there are several crucial aspects that have not been addressed in the current paper, and which certainly have a strong influence on the reported distributions of both the spatial and temporal entropies. On the one hand, the particular tactical patterns of each football team may be responsible for the reported entropies. Players are constrained to move or remain at certain areas of the pitch, and the entropy related to their passes is consequently affected. In that sense, our results are just descriptive and cannot give an explanation about the origins of the reported entropy. The interaction with the opposing team is another factor that constrains the entropy of a team, since the position of passes is not freely selected by players but is a consequence of the spatial distribution of partners and rivals. The existence of a defensive and attacking phases adds further variables to the analysis, since some of the passes can be considered defensive and others attacking, a dichotomy that has not been considered in the current work.

We must also take into account that there are series of events that can also have crucial influences on the entropy of a team and whose interplay should be analyzed in further studies. The score, substitutions, the reorganization of the team based on a change of tactics and the fatigue of players are few examples of different events whose impacts on the entropy of a team are still unknown. Finally, with regard to the passing networks, several aspects can improve their construction and analysis. First, it would be interesting to quantify the importance of passes and translate it into the weight assigned to the links. Second, the construction of Markov matrices based on the structure of passing networks may also be of interest in order to predict the movements of the ball and the amount of randomness in its dynamics.

In view of all, we believe that our results, and further analysis applying concepts of nonlinear dynamics and network science to football datasets, will result in a deeper understanding of the processes occurring in the pitch during a football match and will give coaches and technical staffs of football teams additional (and complementary) information in order to take the best decisions.

## 4. Methods

### 4.1. Definition of Network Metrics

#### 4.1.1. Centroid Coordinates and Dispersion

$X_{CM}$ and $Y_{CM}$ correspond to the *network centroid coordinates*; i.e., the average position of all passes of each temporal network. Specifically, we only consider the position from where passes are sent. Values are given in field coordinates, which, in both axis, range from 0.00 to 100.00. In this way, the center of the field corresponds to coordinates [50.00,50.00] and the center of the opponent’s goal is (100.00, 50.00) (0.00, 50.00) being the center of the team’s own goal). The *centroid dispersion* $\sigma_{CM}$ corresponds to the standard deviation of the average location of players (obtained from their passes) with regard to the position of the network centroid.

#### 4.1.2. Clustering Coefficient

The *clustering coefficient* of a node *i* is obtained as the percentage of the nodes directly connected to it that, in turn, are connected between them. This measure can be averaged along the *N* nodes of a network to obtain the *average clustering coefficient*. However, since passing networks are weighted, we calculated the weighted clustering coefficient $C_{w}\left(i\right)$ to measure the likelihood that neighbors of a given player *i* will also be connected between them [40]:

(1) $$\begin{array}{c} C_{w}\left(i\right)=\frac{\sum_{j,k}w_{ij}w_{jk}w_{ik}}{\sum_{j,k}w_{ij}w_{ik}} \end{array}$$

where *j* and *k* are any two players of the team and $w_{ij}$ and $w_{ik}$ is the number of passes between a third player *i* and the both of them. Finally, the clustering coefficient of the whole network is obtained by averaging $C_{w}\left(i\right)$ over all players; i.e., $C=\frac{1}{N}\sum_{i=1}^{N}C_{w}\left(i\right)$. Note that, the weighted version of the clustering coefficient measures the tendency of a team to form balanced triangles between players.

#### 4.1.3. Shortest Path Length

In a passing network, the *shortest path length* (sp) [41] is the minimum number of players that must be traversed by the ball to go from one player to any other of the team. Since passing networks are weighted (i.e., the number of passes between players is different), we have to take into account the different weights of the links, considering that the higher the weight between two players, the shorter the topological distance between them. Here, the topological length $d_{ij}$ of the link between two players *i* and *j* is defined as the inverse of the link weight, $d_{ij}=1/w_{ij}$. However, when computing sp for weighted networks, the shortest path length between a pair of players may not be a direct link, since there could exist a shorter path by combining two (or more) alternative links. Therefore, we computed the minimal shortest path $p_{ij}$ between all pairs of players using the Dijkstra’s algorithm [42]. Next, we obtained the average shortest path sp of the whole team as:

(2) $$sp=\frac{1}{N(N-1)}\sum_{i,j_{\;\;i\neq j}}p_{ij}$$

where N=11 is the total number of players of the team.

#### 4.1.4. Largest Eigenvalue of the Adjacency Matrix

The *largest eigenvalue* $\lambda_{1}$ of the weighted adjacency matrix *A* of a network is a measure of the network strength [29]. The weighted adjacency matrix *A* is a N×N matrix whose elements $a_{ij}$ contain the number of passes going from player *i* to player *j*. The largest eigenvalue of *A* is bounded by the average number of passes between players 〈S〉, as $\lambda_{1}\geq \langle S\rangle$, and also by $s_{max}\geq \lambda_{1}\geq max\left(\langle S\rangle ,\sqrt{s_{max}}\right)$ [43], where $s_{max}$ is the maximum number of passes that a player has made to any other player of his team. As a rule of thumb, networks with higher number of links (passes) will have a higher $\lambda_{1}$ and networks with the important nodes connected between them (known as assortative networks) will also have higher $\lambda_{1}$ than networks where the hubs (i.e., important players) are not directly connected between them.

#### 4.1.5. Eigenvector Centrality: Maximum Value and Dispersion

The *eigenvector centrality* ec(i) of a player *i* is a measure of node importance that is obtained by calculating the eigenvector $v_{1}$ associated to the largest eigenvalue $\lambda_{1}$ of the weighted adjacency matrix *A*. The eigenvector centrality is a measure of node importance that takes into account the number of all directed connections a player (node) has. Furthermore, two factors contribute to increase the eigenvector centrality of a given node: (i) a higher number of direct connections to other players (note that connections are weighted) and (ii) being connected to other nodes that in turn, also have a high centrality. In this way, important players are those that are strongly connected to other important players of the team. Despite each player having a given eigenvector centrality, it is interesting to compute the standard deviation $\sigma_{ec}$ and highest value $ec_{max}$, the former indicating whether all players participate equally to the construction of the passing network and the latter showing whether there exists a player accumulating a high importance in the team.

#### 4.1.6. 50-Pass Network Time

The *50-pass network time* Δt is the time required to construct a 50-pass network. It is obtained subtracting the time of the first pass of the network from the time of the last pass. Teams with shorter Δt are those that generate more passes in less time. As explained in [13], the number of passes considered to construct the network (n=50) must be high enough to guarantee the construction of a network between players and low enough to avoid the covering of very long periods. As in [13], n=50 is a trade-off value between both limits.

### 4.2. Temporal Entropy and Complexity

**Figure 8:**
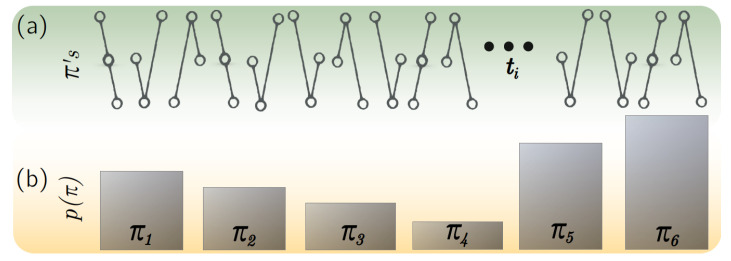
Obtaining ordinal patterns from time series. (**a**) An example of a set of ordinal patterns π of D=3. Some of them are repeated more frequently than others. (**b**) D!=6 different ordinal patterns can be possible for D=3, leading to a probability distribution p(π) with 6 possible elements.

We used a temporal entropy metric that depends on the information content of a random variable $x_{t}$. Here, $x_{t}$ contains the evolution of a network parameter of one team sampled each 50 passes (see Figure 5). We computed the evolution of nine network parameters and computed their temporal entropy $H_{t}$ and statistical complexity $SC_{t}$. Given a finite collection of samples $x_{t}$ of length *M*, one can define an embedding dimension *D* that implicitly defines both the length of the symbol vectors π of a time series and the number of possible patterns as ||{π}||=D!. Consecutive amplitudes of $x_{t}$ of dimension *D* are mapped to a specific π-symbol that lies in an ordinal space of dimension D−1 as follows: the highest value of the *D*-vector of $x_{t}$ is given by the ordinal value of D−1, while the point of the series with lowest amplitude will be transformed into the lowest possible value 0. Intermediate amplitudes are thus organized in the ordinal space between [0,D−1]. For example, assuming D=3, let us suppose that we want to translate the initial D=3 values {0.3,0.01,0.87} of the series into an ordinal pattern. We would obtain an ordinal pattern π equal to (1,0,2). We use the condition M>>D! to statistically guaranty the appearance of all available ordinal patterns in $x_{t}$. Once the first ordinal pattern at time $t_{1}$ is obtained, we proceed with the remaining ordinal patterns by sequentially repeating the previous process upon transforming the whole signal into a symbol sequence $s_{t}$ containing M−D ordinal patterns of length D=3 (see Figure 8a).

Next, we obtained the probability of appearance of each pattern p(π), which was built by taking the normalized histogram of all available π’s on $x_{t}$. The probability of finding a specific pattern π was used to compute the temporal entropy $H_{t}\left[p\left(\pi \right)\right]$. Specifically, $H_{t}$ was the permutation entropy [30] that was taken as the ratio obtained from dividing the Shannon entropy of p(π) by the maximum possible entropy: $H_{t}\left[p\right]=-\sum p_{i}log\left(p_{i}\right)/S_{max}$ with $S_{max}=S\left[p_{e}\right]$ and $p_{e}=\left\{1/D!\right\}$ as a uniform distribution. Note that in said way, the temporal entropy was normalized such that $0\leq H_{t}\leq 1$. $H_{t}$ quantified the levels of order (few patterns available in the signal) and disorder (all patterns are equally available) of a signal. Finally, we computed the statistical complexity SC[p] by means of the statistical disequilibrium Q[p]. Disequilibrium evaluates the existence of preferred patterns among the accessible ones as $Q\left[p\right]=Q_{0}D\left[p,p_{e}\right]$ with $Q_{0}=-2{\left\{\frac{D!+1}{D!}log\left(D!+1\right)-2log\left(2D!\right)+log\left(D!\right)\right\}}^{-1}$ as a normalization constant and D[p,pe] as the Jensen-Shannon divergence between the information content p(π) and the uniform distribution $p_{e}$. Once the disequilibrium was obtained, the statistical complexity was calculated as the product $SC_{t}\left[p\right]=H_{t}\left[p\right]Q\left[p\right]$. High values of the statistical complexity correspond to time series that have a diversity of patterns but, at the same time, they do not follow a random distribution. In the paper, we decided to use D=4 since the length *M* of the time series of networks’ parameters allowed us to arrive to this dimension. 

## Figures and Tables

**Table 1:** Structure of the datasets. Time, in seconds, corresponds to the moment when a pass was made. Player 1 and Player 2 are, respectively, the sender and receiver of the pass, while $x_{1,2}$ and $y_{1,2}$ are the coordinates of both players, in field units (bounded, at both axis, between 0 and 100).

Time (Seconds)	Team	Player 1	$x_{1}$	$y_{1}$	Player 2	$x_{2}$	$y_{2}$
…	…	…	…	…	…	…	…
128	Real Madrid	Ramos	33.47	58.35	Modrić	42.30	58.75
130	Real Madrid	Modrić	59.36	70.00	Benzema	60.10	74.90
136	Real Madrid	Benzema	60.15	80.15	Vinícius	65.40	86.50
…	…	…	…	…	…	…	…

**Figure A1:**
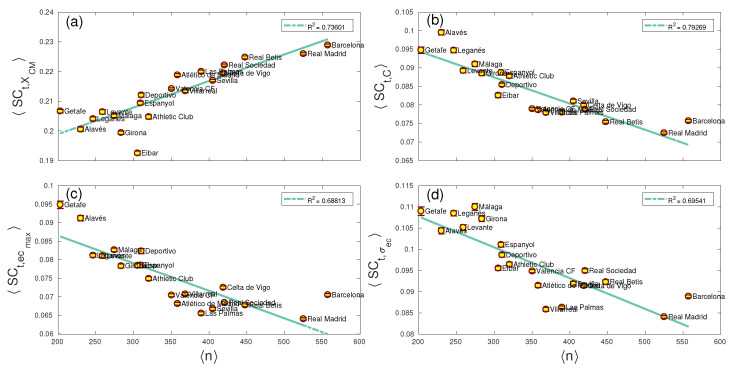
Temporal Complexity $SC_{t}$ vs. average number of 〈n〉. Only network parameters with a correlation $r^{2}>0.6$ are shown; namely, the *x*-coordinate of the network centroid $X_{CM}$ (**a**), the clustering coefficient *C* (**b**), the largest eigenvector centrality of a player $ec_{max}$ (**c**) and the dispersion of the centrality of all players of the team $\sigma_{ec}$ (**d**).

## Appendix A

Figure A1 complements Figure 7, showing those network parameters whose complexity had a correlation with the average number of passes 〈n〉 fulfilling $r^{2}>0.6$. Note that in all cases, complexity behaves in the opposite way to the entropy.
